# Impact Ionization Coefficient Prediction of a Lateral Power Device Using Deep Neural Network

**DOI:** 10.3390/mi14030522

**Published:** 2023-02-23

**Authors:** Jingyu Cui, Linglin Ma, Yuxian Shi, Jinan Zhang, Yuxiang Liang, Jun Zhang, Haidong Wang, Qing Yao, Haonan Lin, Mengyang Li, Jiafei Yao, Maolin Zhang, Jing Chen, Man Li, Yufeng Guo

**Affiliations:** 1 The College of Integrated Circuit Science and Engineering, Nanjing University of Posts and Telecommunications, Nanjing 210023, China; 2 National and Local Joint Engineering Laboratory for RF Integration and Micro-Packaging Technologies, Nanjing University of Posts and Telecommunications, Nanjing 210023, China

**Keywords:** impact ionization coefficient, deep neural networks (DNN), avalanche breakdown, lateral power device

## Abstract

Nowadays, the impact ionization coefficient in the avalanche breakdown theory is obtained using 1-D PN junctions or SBDs, and is considered to be a constant determined by the material itself only. In this paper, the impact ionization coefficient of silicon in a 2D lateral power device is found to be no longer a constant, but instead a function of the 2D coupling effects. The impact ionization coefficient of silicon that considers the 2D depletion effects in real-world devices is proposed and extracted in this paper. The extracted impact ionization coefficient indicates that the conventional empirical impact ionization in the Fulop equation is not suitable for the analysis of 2D lateral power devices. The veracity of the proposed impact ionization coefficient is validated by the simulations obtained from TCAD tools. Considering the complexity of direct modeling, a new prediction method using deep neural networks is proposed. The prediction method demonstrates 97.67% accuracy for breakdown location prediction and less than 6% average error for the impact ionization coefficient prediction compared with the TCAD simulation.

## 1. Introduction

Lateral power devices enable the combination of a good integration and power control capability, and already dominate the power integrated circuit market. As one of the most fundamental performances, the off-state breakdown characteristic of lateral power devices is limited by the impact ionization and the avalanche breakdown process [1,2,3]. As lateral double-diffused metal-oxide-semiconductor transistors (LDMOSs) are typical 2D lateral power devices that rely on 2D coupling effects and structures to sustain a good trade-off between off-state and on-state performances, in order to analytically obtain the breakdown voltage (BV), the accurate impact ionization model and the 2D electric field profile are both required. Yet, the current avalanche breakdown and impact ionization theories were initially approached and interpreted in the context of the 1D structures and models that exclude the impact of the inherent 2D coupling effects in 2D breakdown structures [4,5]. Therefore, the avalanche breakdown voltage of those devices can only be analytically predicted within the particular range of device parameters in the realm in which the devices’ 2D effects are marginal. However, the relationship between the 2D device structure and the device’s avalanche breakdown performance is essential for the design and exploration of lateral power devices. In fact, as the core performance of the power devices is to handle high voltage and power, the 2D coupling effect is much more important in state-of-art lateral power devices as it is the key to realizing an even surface electric field, in order for a high breakdown voltage of the device to be achieved. Because of the missing key impact ionization coefficient for lateral power devices with a strong 2D coupling effect, although accurate analytical models based on 1D/2D Poisson’s equation can be employed so that the 2D electric field and potential distribution can be depicted, the conventional 1D PN junction-based impact ionization model can no longer be utilized to correctly determine the device’s BV [2,3,4,5,6]. Consequently, nowadays, the analytical breakdown voltage models are obtained by combining a 1D/2D electric field/potential model and an empirical critical electric field as a compromise. Such a compromise cripples the role of simple but efficient analytical models for exploring and designing lateral power devices. Therefore, in most cases, TCAD tools are still needed, even if the 2D electric field and potential model have already been analytically obtained [7,8,9,10]. Meanwhile, with advanced lateral power devices possessing more and more complicated 2D structures, the combination of the conventional impact ionization model and finite element analysis in TCAD tools also leads to a significant boost in computation and a much more severe convergence issue.

Because of its modeling ability in coupling latent relationships between the input and output data, the machine learning (ML) techniques that have emerged in recent years provide a potential means to effectively predict the devices’ electrical performance with efficiency and veracity, without involving complicated physical models and finite element analysis [11,12,13,14]. However, the application of ML-based techniques in the power device realm mainly focuses on predicting the devices’ performance. There has been less focus on solving more fundamental device physics issues.

In this paper, in order to accurately obtain the analytical breakdown voltage of lateral power devices, the modified impact ionization coefficient is first proposed in order to demonstrate the influence of 2D effects on impact ionization and avalanche breakdown in silicon-on-insulator (SOI) lateral power devices. This modified impact ionization coefficient differs from the conventional empirical coefficients obtained using a 1D PN junction structure. The modified impact ionization model in this paper considered the 2D coupling effect that resulted from the actual 2D structure of lateral power devices. Consequently, such a modified impact ionization coefficient could handle not only 1D, but also 2D problems. The correctness of the proposed modified impact ionization coefficient was validated using commercial TCAD tools. The breakdown voltage of lateral power devices can be thus accurately be predicted by using simple analytical models without employing the TCAD tools. Considering the complexity of these influences, a deep neural network (DNN)-based extraction method is proposed to accurately and swiftly predict the modified impact ionization coefficient, which is a strong function of the 2D drift region parameters. The results show that the proposed method can achieve less than 6% average prediction error for impact ionization coefficient prediction compared with the device simulation results, while demonstrating a significant speedup.

## 2. Modified Impact Ionization Coefficient

### 2.1. Ionization Coefficient of 2D Drift Region

The avalanche breakdown voltage of a lateral power device is determined by the impact ionization in a semiconductor layer. The impact ionization coefficient is considered to be only affected by the material itself and the electric field profile. Considering the generality of the question and the simplicity of analyzing, the silicon-based lateral power device is demonstrated in this paper. Unlike the silicon carbide (SiC) or gallium nitride (GaN)-based power devices whose avalanche breakdown performance is a strong function of inevitable defects, silicon-based lateral power devices have a more stable avalanche breakdown performance and more authentic TCAD simulation results, and do not require more calibration from experimental results. However, as the avalanche breakdown and impact ionization process are rather fundamental device physics, such a method can also be applied to various types of power devices. However, as mentioned above, due to the strong impact of defect-induced early breakdown, the modeling and characterization of impact ionization in semiconductors such as GaN and SiC are expected to be more complicated.

For a silicon-based power device, according to the classic avalanche breakdown theory, the impact ionization is only a function of the electric field profile and material, and thus the impact ionization coefficient can be obtained using the Fulop equation (an empirical expression), which yields the following:

(1) $$\alpha_{n}=\alpha_{0}\cdot E{\left(x\right)}^{7}$$

here, *a*_0_ is the impact ionization coefficient of silicon, which satisfies *a*_0_ = 1.8 × 10^−35^. It is worth noting that the impact ionization coefficient is an empirical coefficient and is conventionally considered to be only influenced by the material itself. Therefore, the breakdown voltage and critical electric field can be determined by the avalanche breakdown condition, which can be given as follows:

(2) $$\int_{0}^{W}\alpha_{n}dx=1$$

For simplicity, only the impact ionization of the electron is considered, and the impact ionization coefficients of the electron and hole are considered to be the same. Equation (2) indicates that when the device undergoes avalanche breakdown, the impact ionization rate approaches infinity, i.e., the number of electron–hole pairs generated due to impact ionization along a specified current path is infinite and the value of the ionization integral is 1. By employing the classic Fulop equation and the avalanche breakdown condition shown in Equation (2), the avalanche breakdown voltage of a 1D PN junction can be accurately calculated.

**Figure 1:**
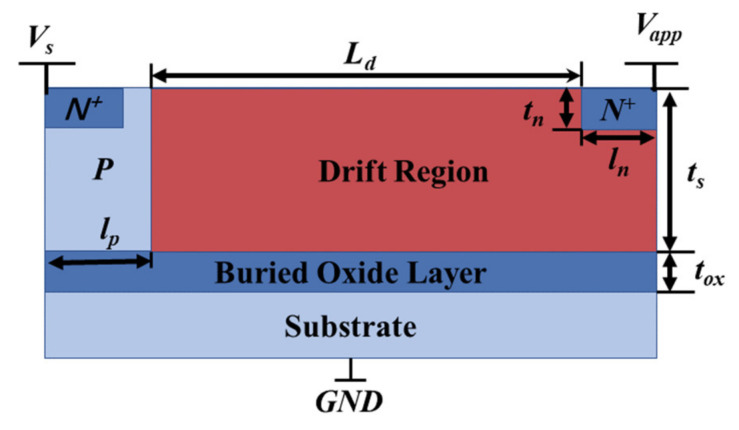
Schematic diagram of a typical SOI lateral power device structure.

**Figure 2:**
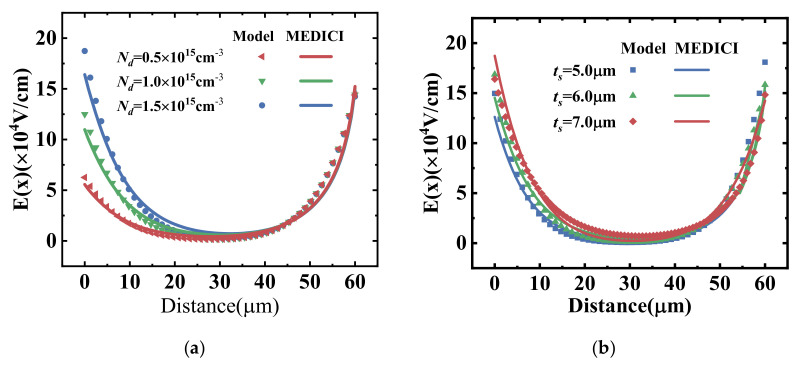
Modeled and simulated surface electric field profile of the SOI S−RESURF lateral power devices with (**a**) the doping concentration of the drift region and (**b**) thickness of the epitaxial layer.

However, for the lateral power devices with 2D coupling effects that cannot be ignored, the avalanche breakdown voltage of the device can no longer be directly solved by using the Fulop equation and analytical electric field profile. A typical silicon-on-insulator (SOI) lateral power device is shown schematically in Figure 1. The SOI layer thickness is *t_s_* and the dielectric constant is *ε_s_*. The buried oxide (BOX) layer thickness is *t_ox_* and the dielectric constant is *ε_ox_*. When the devices are reversed-biased, the lateral power device operates in off-state, in which case the lowly doped drift region sustains most of the applied voltage by depletion. Once the applied voltage exceeds the breakdown voltage, a great number of electron–hole pairs are generated by the impact ionization process, forming a big current between drain and source contacts, and avalanche breakdown occurs. Avalanche breakdown can occur either when the drift region is partially or fully depleted. Yet, for the purpose of device optimization, the practical devices are mostly designed to have avalanche breakdown when full-depletion conduction is reached. Figure 2 shows that the surface electric field distribution for different device structure parameters derived from the virtual graded junction theory and the TCAD simulation tools, and the agreement between the model and the simulation results proves the accuracy of the breakdown model [15,16,17].

**Figure 3:**
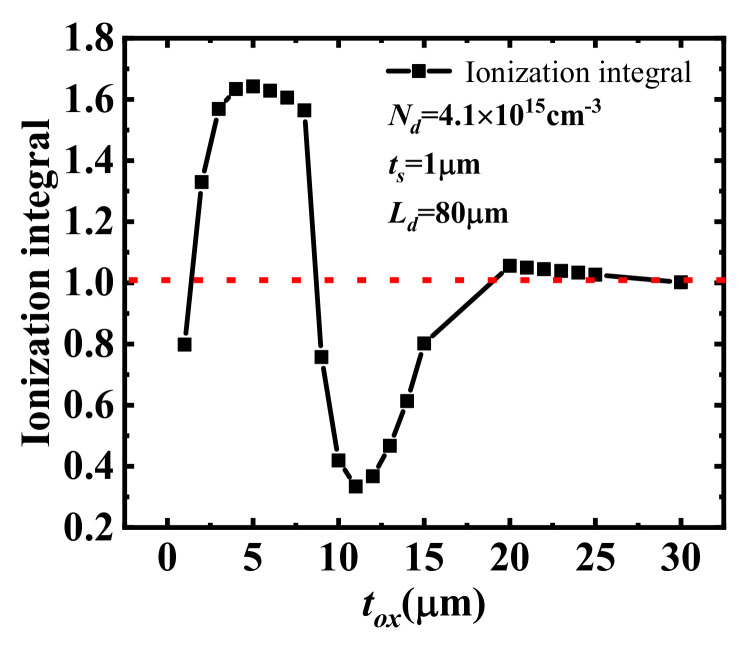
Variation curve of the ionization integral with *t_ox_* for the avalanche breakdown of lateral power devices. The red dashed line shows the trend value of the ionization integral when *t_ox_* increases to a certain level.

Although the surface electric field of the drift region can be depicted by 2D or even 1D analytical models, as shown in Figure 2, the calculated BV obtained by directly solving Equation (2) is far from the simulated and measured BVs. For instance, when the thickness of the buried oxide layer (*t_ox_*) of an SOI lateral power device is relatively small, the 2D coupling effects of the lateral power device are significant. According to the classic avalanche breakdown theory, it is expected that the ionization integral along the surface is equal to 1, or at least near 1, considering the error caused by the analytical model when the lateral breakdown occurs. Yet, by calculating the ionization integral using Equations (1) and (2) under various *t_ox_*, as shown in Figure 3, the ionization integral along the surface can be far above or under 1. Namely, the currently-used impact ionization coefficient is no longer applicable to describe the avalanche breakdown condition of 2D devices. The reason for the large discrepancy between the BV derived from Fulop’s power law and TCAD simulations is the influence of the 2D coupling effect of the lateral power device. As shown in Figure 3, when *t_ox_* increases to a certain level, the 2D coupling effect of the lateral power device is marginal, and thus the ionization integral tends to 1, which is clearly consistent with that in the 1D cases.

**Figure 4:**
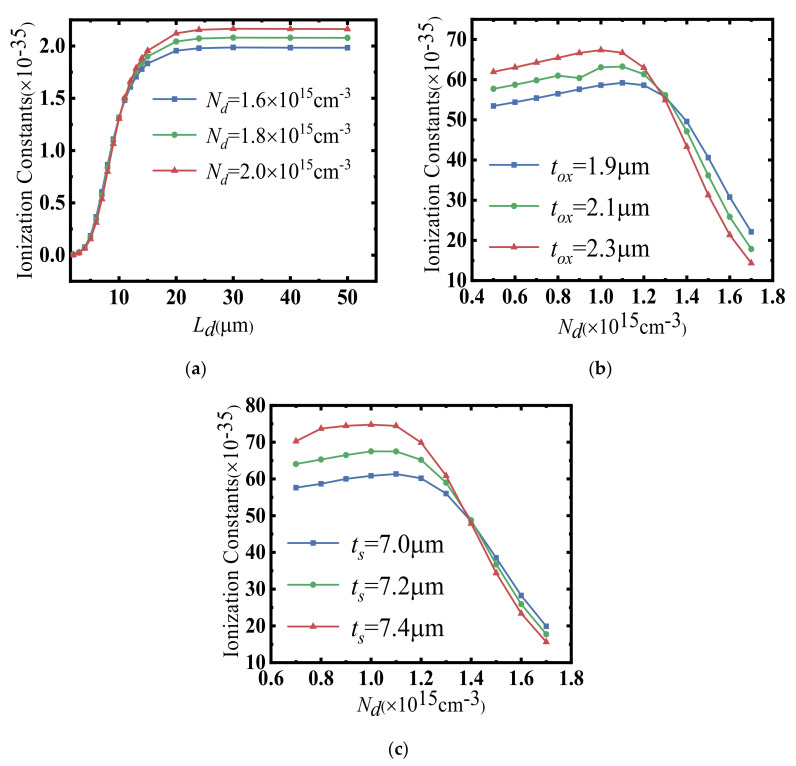
(**a**) Variation curves of *a_0_* with *L_d_* and *N_d_* for different *L_d_* at full depletion of the drift region. (**b**) Variation curves of *a_0_* with *t_ox_* and *N_d_* for different *t_ox_* at full depletion of the drift region. (**c**) Variation curves of *a_0_* with *t_s_* and *N_d_* for different *t_s_* at full depletion of the drift region.

**Figure 5:**
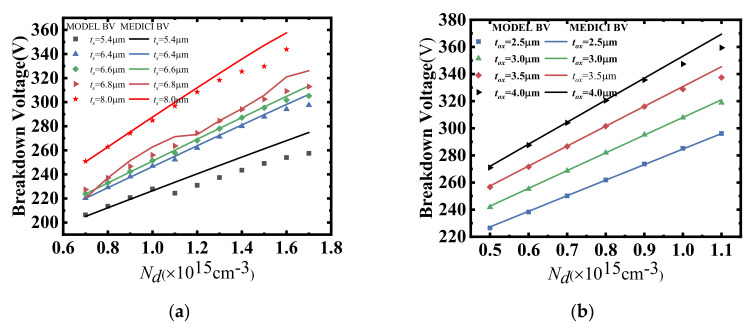
(**a**) Variation curves of BV with *t_s_* and *N_d_* for different *t_s_* at full depletion of the drift region. (**b**) Variation curves of BV with *t_ox_* as and *N_d_* for different *t_ox_* at full depletion of the drift region.

In light of this, the modified impact ionization coefficient for silicon-based lateral power devices is proposed. The extraction of this modified impact ionization coefficient can be achieved by using the combination of virtual graded junction theory and TCAD simulation tools. As shown in Figure 4 and Figure 5, a good agreement between the model and simulation results demonstrates that the empirical model of impact ionization is a guide to accurately solve the avalanche breakdown voltage of 2D lateral power devices.

### 2.2. Empirical Models for the Impact Ionization Coefficient

**Figure 6:**
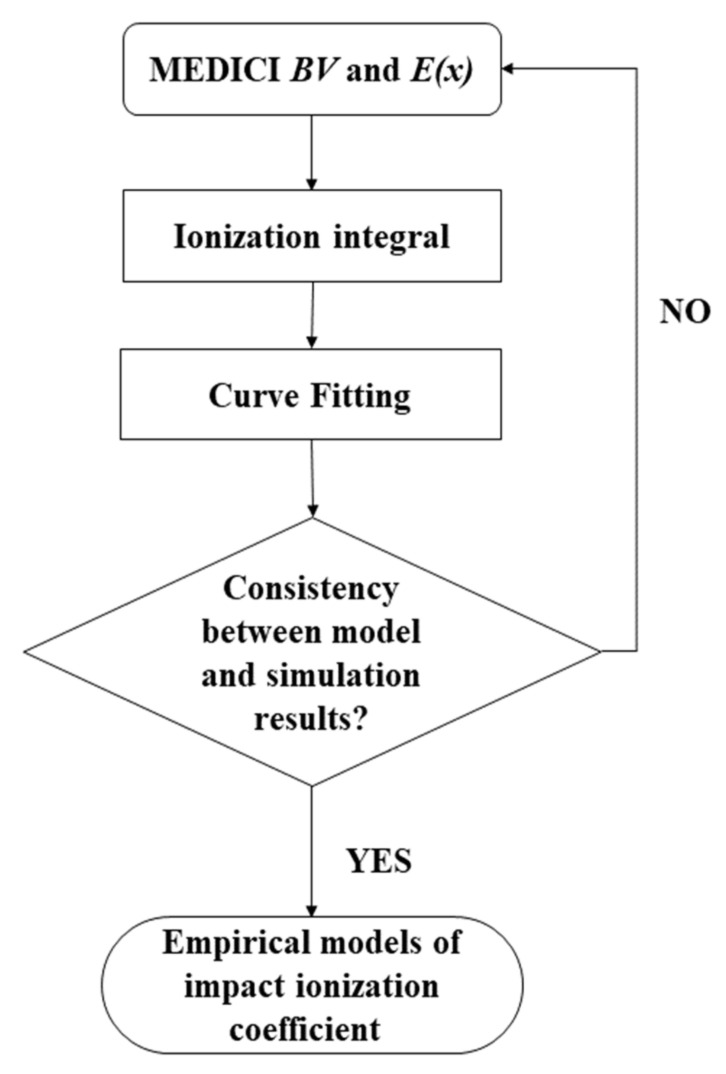
Impact ionization coefficient extraction process.

Figure 6 shows the flowchart for the extraction of the impact ionization coefficients for lateral power devices. The modified impact ionization coefficients are obtained by substituting the surface electric field (*E*(*x*)) and simulated/measured BV into the ionization integral equation. In order to make the ionization integral equal to 1, as the avalanche breakdown condition requires, the modified impact ionization can be given as follows:

(3) $$a_{0}=1/\int_{0}^{L_{d}}E{\left(x\right)}^{7}dx$$

The relationship of the modified impact ionization coefficient *a*_0_ as a function of the device structure parameters can thus be obtained by curve fitting:

(4) $$a_{0}\left(t_{s},N_{d}\right)=\left\{\begin{array}{c} \left(28.956\times t_{s}-122.56\right)\times 10^{-35}\\ \left(4\;\mu m\leq t_{s}\leq 10\;\mu m,0.7\times 10^{15}\;cm^{-3}\leq N_{d}\leq 1\times 10^{15}\;cm^{-3}\right)\\ \left[-\left(81.693+\left(t_{s}-7\right)\times 57.42\right)\cdot N_{d}+171.26+\left(t_{s}-7\right)\times 79.55\right]\times 10^{-35}\\ \left(4\;\mu m\leq t_{s}\leq 10\;\mu m,1.1\times 10^{15}\;cm^{-3}\leq N_{d}\leq 1.7\times 10^{15}\;cm^{-3}\right) \end{array}\right.$$

(5) $$a_{0}\left(t_{ox},N_{d}\right)=\left\{\begin{array}{c} \left(16.9\times t_{ox}+45.7\right)\times 10^{-35}\\ \left(2\;\mu m\leq t_{ox}\leq 5\;\mu m,0.5\times 10^{15}\;cm^{-3}\leq N_{d}\leq 1.0\times 10^{15}\;cm^{-3}\right)\\ \left[-88.77\times N_{d}+184.75-\left(t_{ox}-1.9\right)\times 22.27\right]\times 10^{-35}\\ \left(2\;\mu m\leq t_{ox}\leq 5\;\mu m,N_{d}>1.0\times 10^{15}\;cm^{-3}\right) \end{array}\right.$$

As shown in Figure 5, in order to validate the empirical model of the impact ionization coefficients, we compared the BV derived from the empirical model of the impact ionization with the simulated BV obtained from the TCAD tools. Clearly, a good agreement could be achieved, demonstrating the effectiveness of the proposed methodology. Considering the empirical nature of the impact ionization model and Fulop’s power law, as shown in Figure 5a, the empirical impact ionization model could only accurately depict the modified impact ionization coefficient within a fixed range of device structures. Meanwhile, because of the complicity of the coupling relationship between the device parameters and the modified impact ionization coefficient, the analytical model was too cumbersome and complicated to be derived. Therefore, the prediction method based on the deep neural network (DNN) was introduced in this paper for a more accurate and swift extraction of the modified impact ionization.

## 3. Deep Neural Network for Impact Ionization Coefficient Prediction

As the TCAD simulations and experimental results intuitively demonstrate, the structural parameters related to the drift region have a significant impact on the impact ionization coefficient, including the BOX layer thickness *t_ox_*, drift region concentration *N_d_*, top layer silicon thickness *t_s_*, channel length *L*, cathode *N^+^* length *L_n_* and concentration *N_n_*, anode *P^+^* length *L_p_* and concentration *P_p_*, cathode junction depth *t_n_*, anode junction depth *t_p_*, and the radius of curvature *r*. Hence, the effects related to these structural parameters on the impact ionization coefficient are considered in this work. Table 1 shows the variation range of the structural parameters for predicting the impact ionization coefficient in our proposed DNN approach. It is worth noting that the range listed in Table 1 includes the commonly seen structural parameters for silicon-based lateral power devices.

Because of the complexity of the impact ionization coefficient prediction, the conventional method of the empirical model is no longer applicable considering that the wide range of device parameters may vary. Therefore, the machining-learning-based method is employed to simplify the modeling of impact ionization coefficient. The basic overflow of the proposed modified impact ionization coefficient prediction method is composed of two essential parts, which are the prediction model training and model employment. The DNN model is trained using a group of labeled input and output data in order to establish the inherent relationship between the input and output. Clearly, the key to the modified impact ionization coefficient prediction approach is to form a well-trained neural network. The establishment of the neural network can be divided into two parts. The neural network design is the initial step and the neural network training is the second step. First, the neural network depth (the number of neural network levels), the number of nodes per layer, and the purpose of each node’s activation are required. As a rule, the structure of the network is determined by the complexity of the problem and the number of characteristics. The neural network model’s training makes up the second step. In order to lower the loss function, the weights and biases of the network are modified through backward propagation of the error after the error between the predicted value and the true label is obtained during model training. In this step, the model’s optimization function and loss function are created as well. We then create the framework for the prediction problem.

Clearly, to swiftly and accurately predict the modified impact ionization coefficient, the DNN construction needs to be continuously adjusted to minimize errors based on the training and validation results. In order to correctly annotate large-scale datasets, cross entropy loss is adopted as the loss function [17]. Mathematically, this loss function can be expressed as follows:

(6) $$L\left(Y,Y^{a}\right)=-\frac{1}{n}\overset{n}{\underset{i=1}{\sum }}\left(y_{i}ln\left(y_{i}^{a}\right)+\left(1-y_{i}\right)ln\left(1-y_{i}^{a}\right)\right)\;$$

where *n* is the number of training samples, and *y_i_* and $y_{i}^{a}$ are the actual and predicted results of the output from the *i*th training sample. Moreover, the Adam optimizer is employed to reduce the error as the gradient-descent optimizer for DNN models [18].

**Figure 7:**
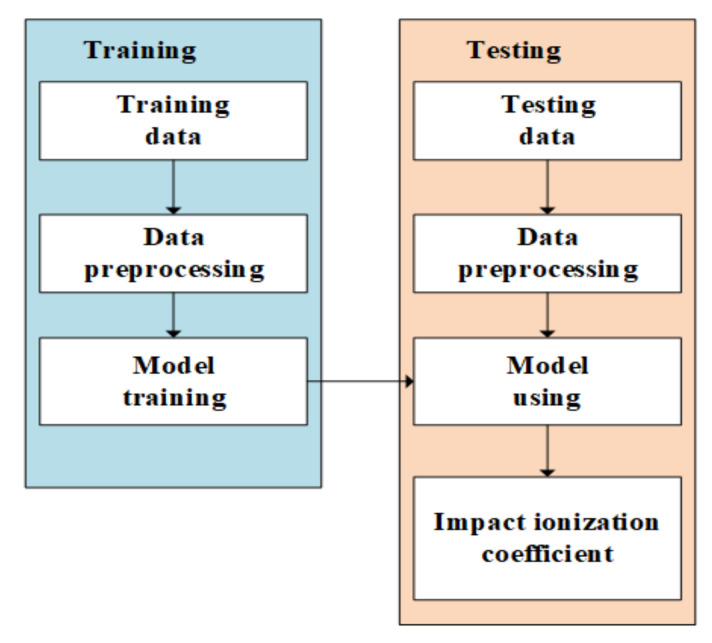
Overview of the machine learning model for impact ionization coefficient prediction.

**Figure 8:**
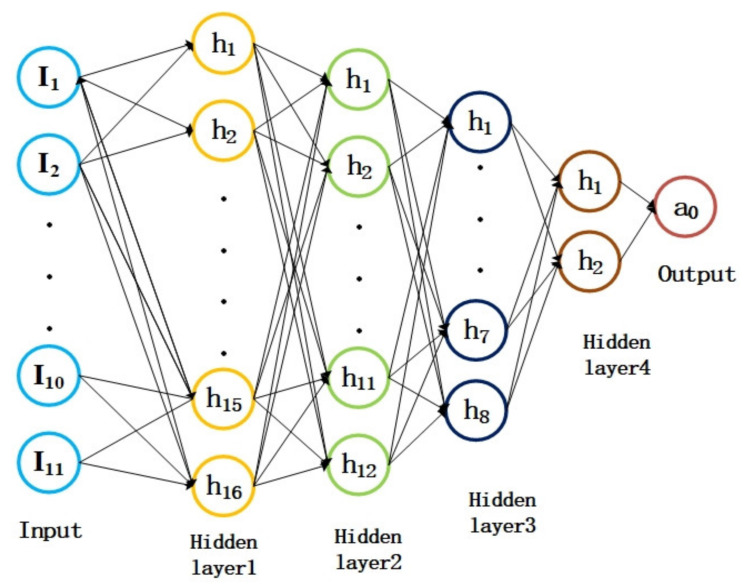
Backpropagation DNN architecture for impact ionization coefficient prediction.

Figure 7 depicts the general process for predicting the impact ionization coefficients using deep neural networks. First, we divide the collected device structure parameters as well as the impact ionization coefficients into two parts: the training data and the test data. The DNN and the training data are then used to train a model for the impact ionization coefficients, and, finally, the test data are used to test the accuracy of the prediction model [19,20,21,22]. As shown in Figure 8, the DNN used in this work consisted of four hidden layers, an input layer, and, finally, an impact ionization coefficient unit. The input layer consisted of the 11 above-mentioned device structure parameters, and the number of neurons in the four hidden layers was adjusted according to the experimental results.

## 4. Simulation and Verification

### 4.1. Dataset and Preprocessing

We collected 1620 sets of data using TCAD simulation tools and MATLAB mathematical tools, which consisted of 11 device structure parameters and the impact ionization coefficient. In addition, 60% of the data were used as the training set and the other 40% as the test set, as shown in Table 2.

There were large order of magnitude differences between the different input data, for example, the doping concentration in the drift region could be up to 10^15^ while the buried oxygen layer thickness was only a few microns. Therefore, in order to improve the accuracy of the prediction and to speed up the gradient descent method to attain the optimal solution, we normalized the input data so that they were all in the range [0, 1].

### 4.2. Predicted Results for the Impact Ionization Coefficient

**Figure 9:**
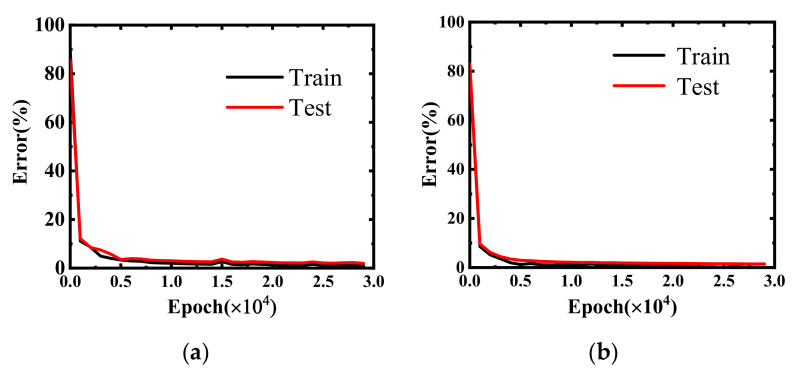
Impact ionization coefficient prediction error during the model training process and testing: (**a**) when the drift region is fully depleted and (**b**) when the drift region is not fully depleted.

**Figure 10:**
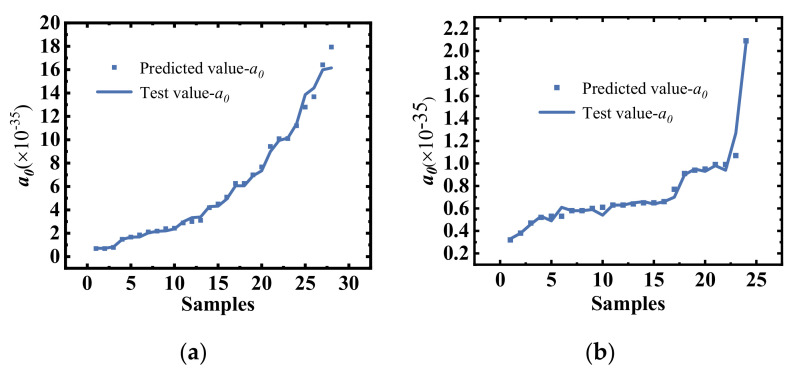
Comparison of the predicted values from the DNN impact ionization coefficient model with test values derived from the TCAD simulation tools: (**a**) when the drift region is fully depleted and (**b**) when the drift region is not fully depleted.

The error curves for the predicted impact ionization coefficients for the training and test sets are shown in Figure 9. The error plot demonstrates the high accuracy and convergence of the impact ionization coefficient prediction model, and the consistency of the training and prediction error curves indicates that the model was trained without overfitting. The high agreement between the predicted and tested values of the impact ionization coefficient is shown in Figure 10, thus demonstrating the high accuracy of the DNN-trained impact ionization coefficient prediction model. The results show that the average error of the impact ionization coefficient was less than 6% compared with the TCAD simulation.

### 4.3. Comparison of Running Times

Table 3 shows the total time taken to run the TCAD numerical simulation tools and the DNN neural network, respectively. It is clear that the use of a deep neural network to predict the impact ionization coefficients will significantly reduce the run time. The simulator’s lengthy prediction time results from the time-consuming procedure it goes through to resolve the associated physical equations using finite element analysis or finite difference methods at set grid points by gradually increasing the applied inverse voltage. Instead, neural networks can be modeled by a machine learning framework based on structural parameters capable of effective prediction based on the model.

## 5. Conclusions

In this paper, we demonstrate the inapplicability of the classic impact ionization coefficient for describing the avalanche breakdown voltage of 2D lateral power devices. To obtain a modified impact ionization coefficient that is applicable to lateral power devices with a 2D coupling effect, a model for predicting the impact ionization coefficient using DNN is presented for the first time. Moreover, DNN is characterized by interpreting complex relationships through neural networks and simplifying complex problems. Thus, this model can be applied to a variety of power devices such as SiC and GaN. With a prediction accuracy of 94%, the prediction model is thus capable of providing guidance when accurately solving the avalanche breakdown voltage of 2D lateral power devices.

## Figures and Tables

**Table 1:** Structural parameters in the drift region.

Structural Parameters	Range
*t_s_*	[0.4, 9] μm
*L_d_*	[10, 135] μm
*N_d_*	[1 × 10^15^, 2 × 10^16^] cm^−3^
*t_ox_*	[0.4, 6.5] μm
*ln*	[2, 14] μm
*N_n_*	[1 × 10^20^, 2 × 10^21^] cm^−3^
*t_n_*	[*t_s_*/3, *t_s_*/1.25] μm
*L_p_*	[3, 15] μm
*P_p_*	[1 × 10^18^, 2 × 10^19^] cm^−3^

**Table 2:** Dataset from TCAD and MATLAB tools.

	Train	Test
**Dataset**	972	648

**Table 3:** Runtime comparison between the TCAD simulator and DNN.

Running Time	MEDICI(s)	DNN(s)
Partial Depletion Case	5.2 × 10^2^	0.01
Full Depletion Case	6.4 × 10^2^	0.03

## Data Availability

Not applicable.
